# Chemical composition and biological activity of *Rubus idaeus* shoots – a traditional herbal remedy of Eastern Europe

**DOI:** 10.1186/1472-6882-14-480

**Published:** 2014-12-12

**Authors:** Mirosława Krauze-Baranowska, Daniel Głód, Marta Kula, Magdalena Majdan, Rafał Hałasa, Adam Matkowski, Weronika Kozłowska, Anna Kawiak

**Affiliations:** Department of Pharmacognosy with Medicinal Plants Garden, Faculty of Pharmacy with Subfaculty of Laboratory Medicine, Medical University of Gdańsk, Gen. J. Hallera Str. 107, 80-416 Gdańsk, Poland; Department of Pharmaceutical Microbiology, Faculty of Pharmacy with Subfaculty of Laboratory Medicine, Medical University of Gdańsk, Gen. J. Hallera Str. 107, 80-416 Gdańsk, Poland; Department of Pharmaceutical Biology and Botany, Wrocław Medical University, Borowska Str. 211, 50-556 Wrocław, Poland; Department of Biotechnology, Division of Plant Protection and Biotechnology, Intercollegiate Faculty of Biotechnology, University of Gdańsk and Medical University of Gdańsk, Kładki Str. 24, 80-822 Gdańsk, Poland

**Keywords:** *Rubus idaeus*, Shoots, HPLC-DAD-ESI-MS, Comprehensive LCxLC, Biological activity

## Abstract

**Background:**

The young shoots of *Rubus idaeus* are traditionally used as a herbal remedy in common cold, fever and flu-like infections yet there is no research concerning this plant material. The aim of the study was to evaluate the chemical composition and biological properties of raspberry shoots from 11 cultivar varieties.

**Methods:**

The methanol extracts were subjected to chromatographic analysis using HPLC-DAD-ESI-MS, and two-dimensional ‘comprehensive’ LCxLC techniques. The biological activity of the shoot extract from the ‘Willamette’ cultivar variety was evaluated. Antioxidant activity was tested using DPPH and phosphomolybdenum assay. Antimicrobial activity was estimated towards 15 strains of human pathogenic bacteria using broth microdilution method. Cytotoxic activity was tested using MTT cell viability assay.

**Results:**

The dominating compounds identified in the shoots of *R. idaeus* were ellagic acid (26.1 - 106.8 mg/100 g) and sanguiin H-6 (139.2 - 633.1 mg/100 g). The best separation of compounds present in the analysed polyphenol complex, was achieved by ‘comprehensive’ LCxLC method using Nucleodur Sphinx RP column in the first dimension and Chromolith Performance column in the second dimension. The shoot extract was found to be a strong antioxidant (EC_50_ 19.4 μg/ml, AAE 427.94 mg/g) and displayed the strongest bactericidal properties towards *Corynebacterium diphtheriae*. The extract revealed higher cytotoxic activity towards the HL-60 cells (IC_50_ 110 μg/ml) than HeLa (IC_50_ 300 μg/ml).

**Conclusions:**

The shoots of *R. idaeus* stand out as a valuable source of sanguiin H-6 and ellagic acid and possess a number of biological properties including antioxidative, antimicrobial and cytotoxic.

## Background

The red raspberry – *Rubus idaeus* L. (*Rosaceae*) is a species widely known for its edible fruits. Although they are most commonly known as food products, they are also a popular anti-inflammatory and antimicrobial remedy used in traditional medicine in eastern parts of Europe. Although the most common herbal drug in folk medicine is the fruit, the shoots of *R. idaeus* have also been used to treat common cold, fever and flu-like infections [1].

Although much interest has been given to the chemical composition and biological properties of raspberries [2], no similar research concerning raspberry shoots was performed up to date. The raspberry fruit contains a number of phenolic compounds, the predominant being anthocyanins and ellagitannins, accompanied by significantly lower concentrations of flavonoids, phenolic acids and flavan-3-ols [2–7]. Ellagitannins are a group of hydrolysable tannins distinctive for the family *Rosaceae*. The main ellagitannin present in *Rubus* species is sanguiin H-6, which is accompanied by lambertianin C and other ellagitannins in small quantities [8, 9]. Ellagitannins, as well as some flavonoids have also been detected in raspberry leaves [10, 11].

HPLC technique coupled with selective detectors, mainly MS [12], is a method of choice in fast determination of the chemical composition of plant extracts [13]. Additionally, two- dimensional HPLC techniques are a new chromatographic approach enabling more efficient resolution of complex samples of plant origin [14].

The aim of this study was to evaluate the chemical composition and biological properties of the shoots obtained from several varieties of raspberries cultivated in Poland.

## Methods

### Plant material

The tops of young, non-lignified shoots of 11 *R. idaeus* cultivar varieties, namely: ‘Benefis’, ‘Beskid’, ‘Glen Ample’, ‘Heritage’, ‘Koral’, ‘Laszka’, ‘Polana’, ‘Polesie’, ‘Poranna Rosa’, ‘Willamette’, ‘Veten’ were collected and identified by Ph. D. Józef Gwozdecki and M. S. Justyna Wójcik from the Department of Pomology, Gene Resources and Nurseries from the Research Institute of Pomology and Floriculture in Skierniewice (Poland). The shoots were dried and powdered. The plants are deposited at the Herbarium of the Medicinal Plants Garden of the Medical University of Gdańsk (Poland) with the following numbers of voucher specimens: 67–2009 (‘Benefis’), 68–2009 (‘Beskid’), 69–2009 (‘Glen Ample’), 70–2009 (‘Heritage’), 71–2009 (‘Koral’), 72–2009 (‘Laszka’), 73–2009 (‘Polana’), 74–2009 (‘Polesie’), 75–2009 (‘Poranna Rosa’), 76–2009 (‘Willamette’), 77–2009 (‘Veten’).

### Sample preparation

The plant material (5 g) was subjected to a continuous, exhausting extraction in a Soxhlet apparatus (100 h) using chloroform and then methanol. The methanol extract was evaporated to the volume of 50 ml and subjected to chromatographic analysis.

For determining biological activity, a dry extract from *R. idaeus* ‘Willamette’ variety was prepared by adding water to the methanol extract, which was then evaporated, lyophilised and stored in airtight containers away from the light.

### Standard compounds

Caffeic acid, chlorogenic acid, ellagic acid, gallic acid, salicylic acid, catechin, hyperoside, quercetin, isoquercetin, kaempferol 3-O-galactoside, myricetin and kaempferol were obtained from Fluka (Switzerland). Procyanidin B_1_, procyanidin B_2_, quercetin 3-O-glucuronide, quercetin 3-O-rhamnoside and tiliroside were obtained from Extrasynthèse (France). Protocatechuic acid, epicatechin and epigallocatechin were obtained from Sigma (Germany). Sanguiin H-6 was isolated according to the previously described procedure [9].

### HPLC system

To evaluate the phenolic content of the shoots of *R. idaeus* a HPLC-DAD-ESI-MS analysis was performed using a HPLC system consisting of steal wash pump LC-20 AD (2), CBM-20 system controller, column termostat CT0-20 AC, auto-sampler SIL 20 AC, detector UV–VIS (Diode Array Detector), mass spectrometer LCMS-2020 with electrospray ionisation (ESI probe), LabSolution computer software (Shimadzu, Japan).

Parameters of MS detector: Detector voltage 3.5 kV, interface voltage 5.0 kV, heat block 200°C, DL temperature 250°C, nebulising gas flow (N_2_) 1.5 L/min, drying gas flow 16 L/min.

### Columns and precolumns

Discovery HS C18 (150 mm × 2.1 mm, 3 μm), Discovery HS C18 (20 mm × 2.1 mm, 3 μm) (pre-column) (Supelco, USA), Nova-pak C 18 (150 × 3.9 mm, 4 μm) (Waters, USA), Nucleodur Sphinx RP (50 mm × 1 mm, 5 μm) (Marcherey-Nagel, Germany), Chromolith Flash RP-18e (25 mm × 4.6 mm), Chromolith Performance (100 mm × 4.6 mm) (Merck, Germany).

### Gradient programs

Solvents: **A -** TFA:water (0.1:100, v/v)**; B -** TFA:water:acetonitrile (0.1:50:50, v/v/v).

I – (percentage of B in A+B): 0 min - 12% B, 10 min - 20% B, 30 min - 43% B, 40 min - 100% B, 55 min - 100% B, 60 min - 12% B, 75 min - 12% B;
II – (percentage of B in A+B): 0 min - 10% B, 70 min - 56% B, conditioning: 74 min - 100% B, 84 min - 100% B, 85 min - 10% B, 95 min - 10% B;
III – (percentage of B in A+B): 0 min – 0% B, 6 min – 0% B, 6.1 min - 10% B, 14 min −10% B, 14.1 min - 15% B, 18 min – 15% B, 18.1 min 17% B, 20 min – 17% B, 20.1 min – 20% B, 22 min – 20% B, 22.1 min – 21% B, 24 min – 21% B, 24.1 min – 23% B, 24, 30 min – 23% B, 30.1 min – 25% B, 32 min – 25% B, 32.1 min – 27% B, 34 min −27% B, 34.1 – 28% B, 36 min – 28% B, 36.1 min – 29% B, 40 min – 29%, 40.1 min - 31% B, 42 min −31% B, 42.1 min – 34% B, 46 min – 34% B, 46.1 min – 35% B, 52 min −35% B, 52.1 min – 45% B, 60 min – 45% B, 60.1 min – 55% B, 70 min – 55% B, conditioning: 70.1 min – 100% B, 84 min −100% B, 84.1 min – 0% B, 95 min – 0%.

### Conditions of one-dimensional HPLC separation

Discovery HS C18 (150 mm × 2.1 mm, 3 μm), Discovery HS C18 (20 mm × 2.1 mm, 3 μm) (pre-column), gradient program I, T = 32°C, v = 0.3 ml/min, v_injection_ = 1 μl, UV λ = 280 nm.

### Conditions of two-dimensional HPLC separation

#### First dimension

Nucleodur Sphinx RP (50 mm × 1 mm, 5 μm) column, gradient program II, T = 20°C, v = 50 μl/min, v_injection_ = 0.4 μl, UV λ = 254 nm.

#### Second dimension

Chromolith Flash RP-18e (25 mm × 4.6 mm), Chromolith Performance (100 mm × 4.6 mm) columns, gradient program III, T = 20°C, v = 6 ml/min, modulation time = 2 min, sampling time = 6.25 Hz, v_loop_ = 50 μl, UV λ = 254 nm. ESI-MS in positive and negative mode.

### Qualitative analysis method validation

The developed HPLC method for purposes of quantitative analysis was validated by determining the calibration curves, linear regression, limit of quantitation (LOQ) and recovery of analysed compounds, which were estimated according to the guidlines of Validation of Chromatographic Methods by Food and Drug Administration, Center for Drug Evaluation and Research http://www.fda.gov/downloads/Drugs/Guidances/UCM134409.pdf (Table 1).

**Table 1:** **Validation parameters for the HPLC method for quantitative analysis of phenolic compounds in the shoots of** ***R. idaeus***

Compound	Calibration curve	r ^2^	LOQ [μg/ml]	Recovery
Gallic acid	y = 0.00022x	0.9998	3.6	110.13 ± 3.86
Chlorogenic acid	y = 0.000265x + 1.666	0.99972	4.2	107.44 ± 5.76
Catechin	y = 0.000409x-0. 222	0.99955	5.7	94.83 ± 4.98
Epicatechin	y = 0.000331848x - 1.007753	0.99995	2.6	101.25 ± 6.02
Isoquercetin	y = 0.000485465x - 0.0266931	0.99997	1.25	103.07 ± 3.98
Ellagic acid	y = 0.000300x + 1.909	0.99964	2.32	102.85 ± 2.98
Hyperoside	y = 0.000413836x - 0.29958	0.99995	1.5	105.62 ± 2.43
Procyanidin B_1_	y = 0.000711836x - 2.04033	0.99996	17.1	105.67 ± 4.15
Procyanidin B_2_	y = 0.000896x - 2.34	0.9999	12.6	96.42 ± 3.58
Sanguiin H-6	y = 0.00109921x + 4.56879	0.99997	7.1	95.82 ± 5.23

r^2^ - linear regression; LOQ - limit of quantitation.

The calibration curves were determined for the standard compounds. Linearity for the working concentrations of the standard compounds was evaluated by determining the correlation coefficient. Stock solutions of standard compounds were diluted in methanol (1 mg/ml). Regression curves were determined basing on the analysis of plot of peak area for the following concentrations of compounds (μg/ml):

– gallic acid and chlorogenic acid: 6.25, 12.5, 25, 50, 100
– catechin, epicatechin, isoquercetin: 3.125, 6.25, 12.5, 25, 100
– ellagic acid, hyperoside: 3.125, 6.25, 12.5, 25, 50, 100
– procyanidin B_1_ and B_2_: 10, 20, 40, 80, 150, 300
– sanguiin H-6: 62.5, 125, 250, 500, 1000.

Regression equations and correlation coefficients are presented in Table 1. LOQ was established as the concentration of the standard compound equalling 10× of the baseline noise. Recovery was determined through adding the standard compounds in the quantities corresponding 50%, 75% and 125% of their content in the plant material, and calculating the percentage of recovery from the median sum of compounds in the plant material as well as the added quantities of standard compounds. Concentrations of quercetin 3-O-glucuronide and an unknown quercetin pentoside were calculated on isoquercetin (Table 1).

### Free radical scavenging ability (FRS) in DPPH assay

The experimental procedure was performed according to Brand-Williams et al. [15]. 150 μl of *R. idaeus* ‘Willamette’ extract was mixed in a 96-well plate (Greiner, Germany) with 0.2 mM methanol solution of DPPH (Sigma). The concentrations of the extract in the reaction mixture were the following: 1, 2.5, 5.0, 10, 25.0, 50.0, 100, 250, and 500 μg/ml. The disappearance of DPPH was monitored spectrophotometrically at 517 nm using microQuant microplate reader (Biotek, USA), during 30 min incubation at room temperature. Free radical scavenging capacity (FRS) was calculated by the following equation: FRS(%) = (100 − ABS_sample_/ABS_DPPH_) × 100, where Abs sample = Abs measured - Abs control (i.e., absorbance of the sample tested without DPPH). From the obtained values, the dose response curve was created, using nonlinear regression module of GraphPad Prism software, followed by calculation of the EC_50_ (defined as the concentration of sample at which 50% of maximum scavenging activity was recorded).

### Reducing power using phosphomolybdenum assay

The modified method of Prieto et al. was used [16]. The shoot extract (200 μl) was mixed with the reagent solution (1.8 ml) containing ammonium molybdate (4 mM), sodium phosphate (28 mM) and sulfuric acid (600 mM). The final tested concentrations were the following: 2.5, 5.0, 10.0, 25.0, 50.0 μg/ml. The reaction mixture was incubated in a water bath shaker at 90°C for 90 min. After cooling, the absorbance of the green phosphomolybdenum complex was measured at 695 nm against a blank (where the extract was replaced by 50% aqueous methanol). The reducing power was compared to the standard antioxidant – ascorbic acid and expressed as ascorbic acid mass equivalents (AAE) (mg/g). The reducing power is calculated as linear dose response slope ratio of extract and ascorbic acid: RP = slope-sample/slope-ascorbic acid.

### Test microorganisms

#### Gram-positive bacteria

β-hemolytic *Streptococcus* group A,B,G, *Streptococcus pneumoniae* (clinical isolates), *Corynebacterium diphtheriae*, *Enterococcus faecalis* (collection of the Department of Pharmaceutical Microbiology, Medical University of Gdańsk), *Staphylococcus aureus* ATCC9027, *Staphylococcus epidermidis* ATCC14990, *Bacillus subtilis* ATCC6633, *Clostridium sporogenes* PCM2486.

#### Gram-negative bacteria

*Klebsiella pneumoniae* (clinical isolate), *Neisseria meningitidis* PCM2586, *Moraxella catarrhalis* PCM2340, *Haemophilus influenzae* PCM2340, *Helicobacter pylori* ATCC10231. Clinical isolates were obtained from St. Adalbert Specialist Hospital in Gdańsk (Independent Public Health Care Facility in Gdańsk, Poland).

### Antibacterial assay

Bacterial cultures were prepared in accordance with literature data by transferring cells from the stock cultures to tubes with adequate broth [17–20], and incubated for 24–48 hours at 37°C. The cultures were diluted to an optical density corresponding to 10^5^ colony forming units per ml (CFU/ml). For *H. pylori*, the inoculum was prepared from colonies grown on TSA (Becton Dickinson, USA) supplement with 5% sheep blood agar plates with final concentration of approximately 10^5^ CFU/ml [21].

Minimum inhibitory concentration (MIC) was determined by broth microdilution technique using 96-well plates. Dry shoot extract was dissolved in water to concentration of 120 mg/ml. Each well was filled with 100 μl of broth, and the shoot extract was added to the wells and diluted in a geometric progression by transferring 100 μl of the solution to the next well (concentrations from 120 to 0.06 mg/ml), followed by adding the microbial suspensions (100 μl) of the tested bacterial strains to each well. Ampicillin was used as a reference compound. The plates were incubated in the conditions appropriate for each bacterium [17–20].

After incubation a visual observation of growth was performed. The MIC was established as the lowest sample concentration that prevented visible growth [22]. In addition 100 μl of suspension from each well without visible growth was inoculated (48 hours) on an agar plate to check bacterial viability. MBC (minimal bactericidal concentration) was defined as the minimum concentration of extract required to kill the bacteria in the medium. For determining *Helicobacter pylori* viability Christiansen broth (home-made, urease test 50 μl) was used.

### MTT cell viability assay

The cytotoxic assay was conducted using human dermal fibroblasts, human promyelocytic leukemia cell line (HL-60) (Department of Drug Technology and Biochemistry, Technical University of Gdańsk, Poland) and human cervical cancer cell line (HeLa) (Department of Histology and Immunology, Medical University of Gdańsk, Poland).

The viability of the cells was determined using the MTT assay. The cells were transferred to 96 well plates, in concentration of 10^5^/well, and incubated overnight (T = 37°C) in the presence of 5% CO_2_. Cells were then treated for 24 hours with *R. idaeus* ‘Willamette’ shoot extract (0–500 μg/ml). MTT (3*-*(4,5*-*dimethylthiazol*-*2*-*yl)*-*2,5*-*diphenyltetrazolium bromide) was added directly to the medium (1,2 mM) and cells were further incubated for 3 hours, followed by DMSO lysis. The absorbance of the formazan solution was measured at λ = 570 nm with a plate reader [23]. The values were then compared with control groups and survivability was calculated from the following equation: survivability percentage = (A_sample_–A_background_)/(A_control_–A_background_) × 100, A-absorbance, − and half maximal inhibitory concentration IC_50_ was calculated.

### Data analysis

For dose response curve fitting in the DPPH assay and linear function formula of dose response in phosphomolybdenum assay analysis of variance (ANOVA), Tukey’s post-choc test were performed (GraphPad Prism 5 Pad Software Inc, La Jolla, USA).

## Results and discussion

### HPLC-DAD-ESI-MS analysis

**Figure 1:**
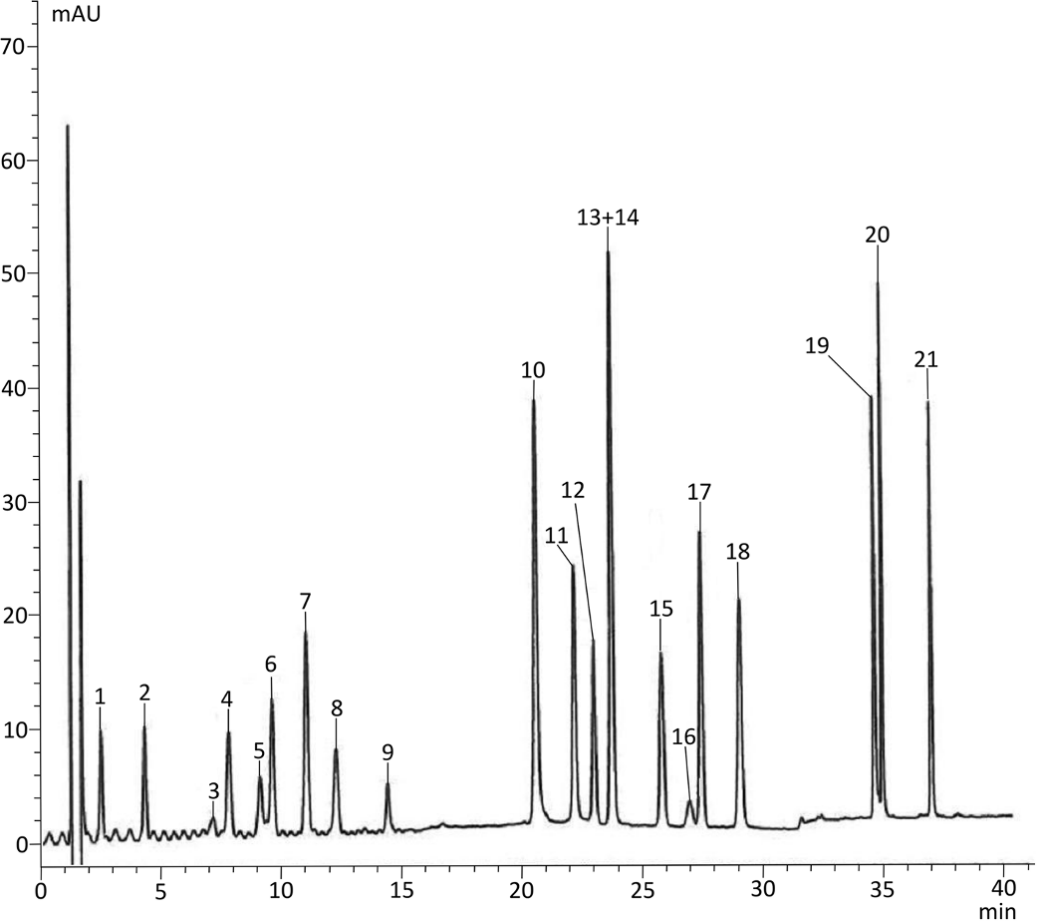
**HPLC chromatograms of standard compounds. 1** – gallic acid, **2** – protocatechuic acid, **3** – procyanidin B_1_, **4** – epigallocatechin, **5** – catechin, **6** – chlorogenic acid, **7** – caffeic acid, **8** – procyanidin B_2_, **9** – epicatechin, **10** – sanguiin H-6, **11** – ellagic acid, **12** – hyperoside, **13** – isoquercetin, **14** – quercetin 3-O-glucuronide, **15** – kaempferol 3-O-galactoside, **16** – salicylic acid, **17** – quercetin 3-O-rhamnoside, **18** – myricetin, **19** – tiliroside, **20** – quercetin, **21** – kaempferol.

In preliminary HPLC-DAD-ESI-MS separations of the mixture of 21 standard compounds, a Nova-pak C 18 column (150 × 3.9 mm, 4 μm) and several gradient elution programmes, varying in gradient profiles and concentration of a mixture of TFA:water:acetonitrile (0.1:50:50, v/v/v) in 0.1% TFA aqueous solution, at different t_G_ values, were used. However, it was not possible to obtain resolution of all standard compounds. The best separation of 20 from the 21 standards (isoquercetin and quercetin 3-O-glucuronide not resolved) was achieved with Discovery HS C18 column connected to Discovery HS C18 precolumn and gradient elution program I, of an increasing concentration of a mixture of TFA:acetonitrile:water (0.1:50:50 , v/v/v), from 12% to 43%, in a 0.1% TFA aqueous solution (Figure 1).

**Figure 2:**
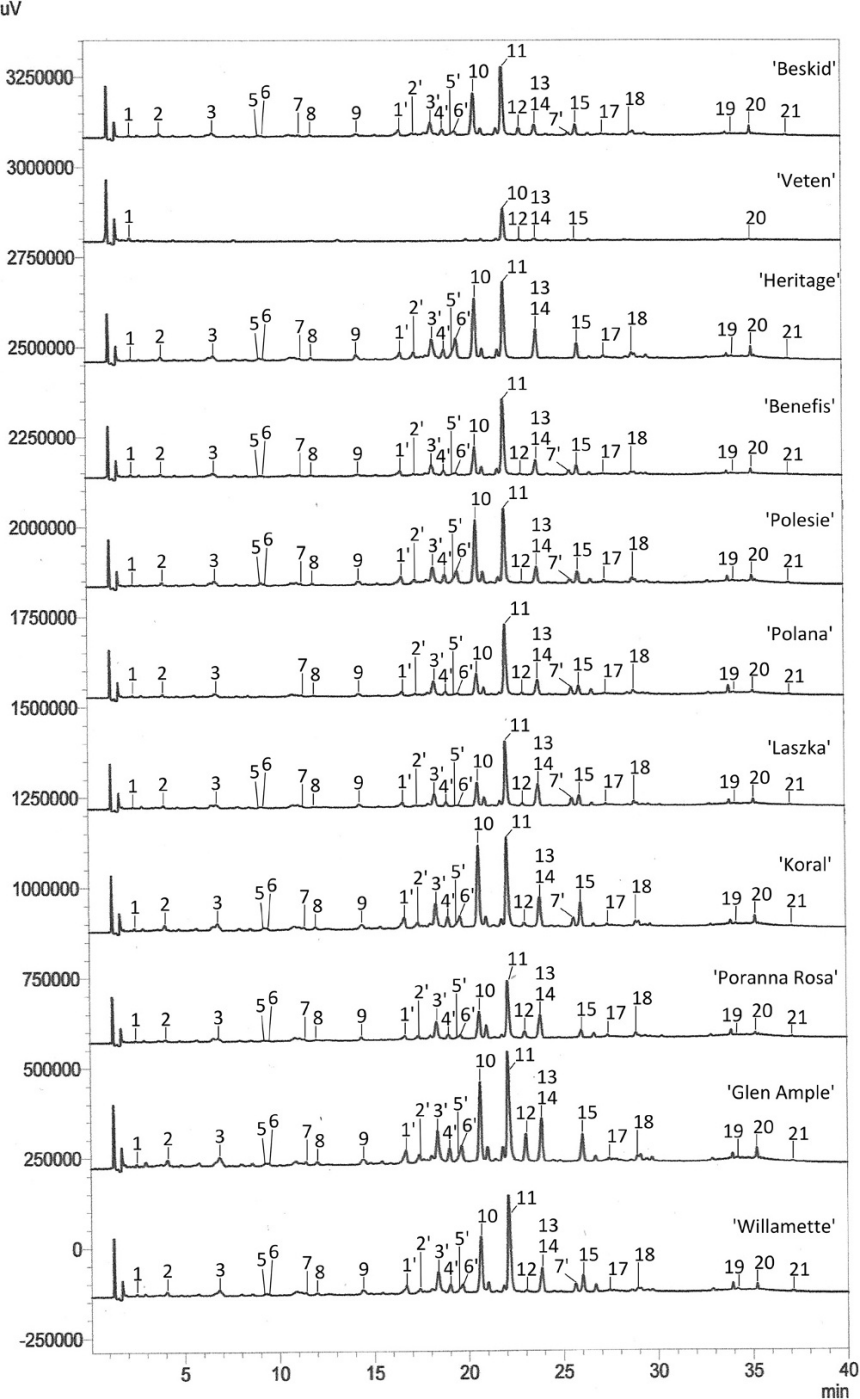
**HPLC chromatograms of 11 varieties of** ***R. idaeus.*** **1** – gallic acid, **2** – protocatechuic acid, **3** – procyanidin B_1_, **5** – catechin, **6** – chlorogenic acid, **7** – caffeic acid, **8** – procyanidin B_2_, **9** – epicatechin, **10** – sanguiin H-6, **11** – ellagic acid, **12** – hyperoside, **13** – isoquercetin, **14** – quercetin 3-O-glucuronide, **15** – kaempferol 3-O-galactoside, **17** – quercetin 3-O-rhamnoside, **18** – myricetin, **19** – tiliroside, **20** – quercetin, **21** – kaempferol, **1′** – sanguiin H-10, **2′** – lambertianin C, **3′** – potentilin/casuarictin, **4′** – sanguiin H-10, **5′** – lambertianin C, **6′** – sanguiin H-2, **7′** – unknown quercetin pentoside. *Discovery HS C18 (150 mm × 2.1 mm × 3 μm), gradient program I.*

Applying the conditions of optimised HPLC-DAD-ESI-MS method, the methanol shoot extracts from 11 cultivar varieties of *R. idaeus* were analysed (Figure 2). Phenolic compounds were identified by comparing their UV spectra and retention time values (t_R_) to that of the standard compounds and by comparison of their mass spectra with literature data (Table 2) [24–29].

**Table 2:** **HPLC-DAD-ESI-MS data of standards and the compounds identified in the shoots of 11 cultivar varieties of** ***R. idaeus***

	Compound	t _R_ (min)	λ _max_ (nm)	Molecular ion [M+H] ^+^/[M-H] ^−^ (m/z)	Fragment ions (m/z)
**1**	Gallic acid	2.4	214, 268	171^+^, 169^−^	-
**2**	Protocatechuic acid	4.2	258, 293	155^+^, 153^−^	-
**3**	Procyanidin B_1_	7.1	278	579^+^, 577^−^	-
**4**	Catechin	9.2	278	291^+^, 289^−^	-
**5**	Chlorogenic acid	9.8	295, 327	355^+^, 353^−^	-
**6**	Caffeic acid	11.0	294, 321	181^+^, 179^−^	-
**7**	Procyanidin B_2_	12.1	278	579^+^, 577^−^	-
**8**	Epicatechin	14.4	277	291^+^, 289^−^	-
**9**	Sanguiin H-6	20.5	251	1869^−^	[934]^−2^, [935]^−2^
**10**	Ellagic acid	22.2	252, 369	303^+^, 301^−^	-
**11**	Hyperoside	23.0	253, 350	465^+^, 463^−^	303^+^, 301^−^
**12**	Isoquercetin	23.8	254, 352	465^+^, 463^−^	303^+^, 301^−^
**13**	Quercetin 3-O-glucuronide	23.8	255, 352	479^+^, 477^−^	303^+^, 301^−^
**14**	Kaempferol 3-O-galactoside	25.9	264, 345	449^+^, 447^−^	287^+^, 285^−^
**15**	Quercetin 3-O-rhamnoside	27.4	254, 348	449^+^, 447^−^	303^+^, 301^−^
**16**	Myricetin	29.0	252, 369	319^+^, 317^−^	-
**17**	Tiliroside	34.5	266, 313	595^+^, 593^−^	-
**18**	Quercetin	35.0	253, 369	303^+^, 301^−^	-
**19**	Kaempferol	37.1	262, 362	287^+^, 285^−^	-
**1′**	Sanguiin H-10	16.7	251	1567^−^	-
**2′**	Lambertianin C	17.4	251	-	[1401]^−2^, [1402]^−2^
**3′**	Potentilin/casuarictin	18.4	251	935^−^	-
**4′**	Sanguiin H-10	19.0	251	1567^−^	-
**5′**	Lambertianin C	19. 5	251	-	[1401]^−2^, [1402]^−2^
**6′**	Sanguiin H-2	19.7	251	1103^−^	-
**7′**	Unknown quercetin pentoside	25.5	254, 353	435^+^, 433^−^	303^+^, 301^−^

t_R_ - retention time; λ_max_ - maximum observed absorbance.

The dominant compounds present in the analysed shoots of *R. idaeus* were ellagic acid and sanguiin H-6 while the other compounds – phenolic acids, flavonoids and flavan-3-ols occurred in much lower concentrations (Figure 2).

From phenolic acids, the presence of gallic acid, protocatechuic acid, chlorogenic acid, caffeic acid and ellagic acid was revealed. Among the chromatographically identified flavonoids, hyperoside, quercetin 3-O-glucuronide, isoquercetin, kaempferol 3-O-galactoside, myricetin, tiliroside, quercetin 3-O-rhamnoside and kaempferol were recognised. The identified flavan-3-ols comprised monomeric catechin and epicatechin, as well as dimeric proanthocyanidins – procyanidin B_1_ and B_2_ (Figure 2, Table 2). These compounds have been previously identified in red and black raspberry fruits, as well as raspberry leaves [2–7, 10, 11].

Since the separation of isoquercetin and quercetin 3-O-glucuronide was not possible in the optimised HPLC conditions, both compounds were identified by the SIM technique. Moreover, by using the SIM technique, the peak observed at t_R_ 25.5 min was tentatively identified as an unknown quercetin pentoside (Table 2). Peaks observed at t_R_ 16.7 – 19.7 min were identified as ellagitannins. In accordance with UV spectra (λ_max_ 251 nm) and m/z values of deprotonated molecules the peaks were identified as: two lambertianin C isomers (t_R_ = 17.4 min and 19.5 min), sanguiin H-2 (t_R_ =19.7 min), two sanguiin H-10 isomers (t_R_ = 16.7 min and 19.7 min) and casuarictin*/*potentillin (t_R_ = 18.4 min) (Figure 2, Table 2). These ellagitannins have been previously described as constituents of raspberry fruits and leaves, as well as in other species from the genus *Rosaceae* [24–29].

### Quantitative analysis of polyphenols in raspberry shoots

Quantitative analysis of polyphenols in the shoots of the 11 *R. idaeus* cultivar varieties was performed using a developed one-dimensional HPLC method (Table 3). Sanguiin H-6 proved to be the dominant polyphenol compound in the shoot extracts with concentrations ranging from 139.2 mg/100 g for ‘Polana’ variety to 633.1 mg/100 g of dry weight for ‘Koral’ variety. These values are similar to those observed for raspberries [9]. The analyzed shoots also contained considerable amounts of free ellagic acid (26.1 – 106.8 mg/100 g), and the determined values were much more differentiated and often higher than in the fruits, where the mean value was about 32.6 mg/100 g (unpublished results).

**Table 3:** **Concentrations of selected polyphenols in the 11 varieties of** ***R. idaeus*** **shoots (mg/100 g dry weight) (n = 3)**

***R. idaeus***variety	Sanguiin H-6	Ellagic acid	Epicatechin	Hyperoside	Isoquercetin and quercetin 3-O-glucuronide	Quercetin pentoside*	Polyphenol sum
Willamette	489.8 ± 50.8	106.8 ± 10.9	73.5 ± 7.4	5.0 ± 0.5	67.4 ± 6.8	23.9 ± 2.5	**766.4**
Poranna Rosa	199.6 ± 20.7	48.4 ± 4.9	50.0 ± 5.0	14.7 ± 1.5	56.7 ± 5.7	-	**369.4**
Glen Ample	394.6 ± 40.9	77.3 ± 1.9	42.4 ± 4.3	32.2 ± 3.2	47.3 ± 4.7	-	**593.2**
Koral	633.1 ± 65.6	80.4 ± 8.2	52.0 ± 5.2	9.7 ± 1.0	58.0 ± 5.8	x	**833.1**
Laszka	170.9 ± 17.7	41.3 ± 1.0	30.9 ± 3.1	5.2 ± 0.5	42.7 ± 4.3	x	**291.0**
Polana	139.2 ± 14.4	55.8 ± 5.6	42.9 ± 4.3	3.3 ± 0.3	36.6 ± 3.7	x	**277.7**
Polesie	523.6 ± 54.2	62.9 ± 6.3	32.7 ± 3.3	x	29.6 ± 3.0	x	**648.9**
Benefis	195.8 ± 20.3	71.1 ± 7.1	16.6 ± 1.7	4.1 ± 0.4	36.0 ± 3.6	x	**323.6**
Heritage	481.1 ± 49.8	63.6 ± 6.4	85.3 ± 8.6	-	55.5 ± 5.6	-	**685.4**
Veten	x	26.1 ± 2.6	10.9 ± 1.1	x	10.3 ± 1.0	x	**47.3**
Beskid	347.4 ± 35.9	56.6 ± 5.7	23.1 ± 2.3	12,67 ± 1,27	14.5 ± 1.5	x	**454.2**

*calculated as for isoquercetin; x – below the limit of detection.

The content of epicatechin was established between 10.9 mg/100 g (‘Veten’) – 85.3 mg/100 g (‘Heritage’), and 3.3 mg/100 g (‘Polana’) – 32.2 mg/100 g (‘Glen Ample’) for hyperoside. Isoquercetin and quercetin 3-O-glucuronide could not be fully separated in the optimized HPLC-DAD-ESI-MS conditions and their concentrations are presented as a sum (content from 10.3 mg/100 g for ‘Veten’ to 67.4 mg/100 g for ‘Willamette’).

The shoots of ‘Koral’, ‘Polesie’, ‘Willamette’ and ‘Heritage’ cultivar varieties proved to be the richest source of sanguiin H-6 (481.1 mg/100 g – 633.1 mg/100 g). Ellagic acid was present at the highest concentrations in ‘Willamette’, ‘Koral’, ‘Glen Ample’ and ‘Benefis’ cultivar varieties (106.8 mg/100 g – 71.1 mg/100 g). The two flavonoids, isoquercetin and quercetin 3-O-glucuronide, were found at the highest concentrations in the shoots of ‘Willamette’, ‘Koral’, ‘Poranna Rosa’ and ‘Heritage’ varieties (67.4 mg/100 g – 55.5 mg/100 g). Most of the shoots contained hyperoside amounts between 3.3 and 14.7 mg/100 g, with the exception of ‘Glen Ample’ variety where the content was much higher (32.2 mg/100 g). Unidentified quercetin pentoside was present at the highest concentration in the shoots of ‘Willamette’ cultivar (23.9 mg/100 g) while most of the other varieties contained levels below the limit of detection. Gallic acid, chlorogenic acid, catechin, procyanidin B_1_ and B_2_ were present below the limit of detection. In the stems of ‘Willamette’, ‘Koral’, ‘Polesie’ and ‘Heritage’ varieties overall sum of polyphenols was several times higher than the other varieties (648.87 mg/100 g – 833.11 mg/100 g) (Table 3).

### Quantitative analysis of polyphenols in *R. idaeus*‘Willamette’ dry shoot extract

The results of the quantitative analysis of the dry shoot extract from *R. idaeus* ‘Willamette’ are presented in Table 4. The extract contained about 5% of sanguiin H-6 and about 1% of free ellagic acid, which is approximately 10 times higher than in the dried shoots (Table 3). It also contained high amounts of epicatechin, isoquercetin, quercetin 3-O-glucuronide and procyanidins B_1_ nad B_2_ (Table 4).

**Table 4:** **Concentration of selected polyphenols in the** ***R. idaeus*** **‘Willamette’ dry shoot extract (mg/100 g dry weight) (n = 3)**

Compound	***R. idaeus***‘Willamette’
Sanguiin H-6	5256.0 ± 469.5
Ellagic acid	1151.7 ± 102.9
Epicatechin	791.7 ± 70.7
Isoquercetin and quercetin 3-O-glucuronide	717,57 ± 64,1
Procyanidin B_2_	646.0 ± 57.7
Procyanidin B_1_	299.0 ± 26.7
Quercetin pentoside	252.0 ± 22.5
Chlorogenic acid	177.4 ± 15.9
Catechin	129.3 ± 11.6
Gallic acid	72.2 ± 6.5
Hyperoside	52.3 ± 4.7
Polyphenol sum	9545.2

The dry shoot extract proved to be richer in phenolic compounds than the raw plant material and was chosen for biological activity analysis.

### Separation of *R. idaeus*shoot polyphenols by ‘comprehensive’ LCxLC two-dimensional liquid chromatography

**Figure 3:**
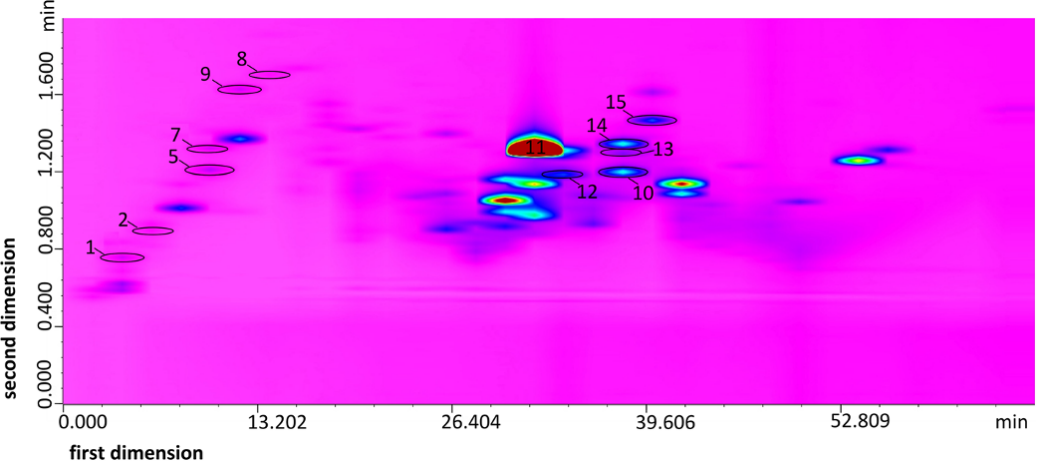
**LCxLC chromatogram of the methanol extract from** ***R. idaeus*** **‘Willamette’ shoots. 1** – gallic acid, **2** – protocatechuic acid, **5** – catechin, **7** – caffeic acid, **8** – procyanidin B_2_, **9** – epicatechin, **10** – sanguiin H-6, **11** – ellagic acid, **12** – hyperoside, **13** – isoquercetin, **14** – quercetin 3-O-glucuronide, **15** – kaempferol 3-O-galactoside. ***first dimension –*** *Nucleodur Sphinx RP (50 mm × 1 mm × 5 μm), gradient program II,* ***second dimension –*** *Chromolith Performance (100 mm × 4.6 mm), gradient program III, UV λ = 254 nm.*

To achieve better resolution and to separate isoquercetin and quercetin 3-O-glucuronide a ‘comprehensive’ two-dimensional LCxLC method was developed (Figure 3). Two-dimensional HPLC is a technique that allows greater values of peak capacity (P) and therefore provides high usefulness in the analysis of complex plant samples [14]. *R. idaeus* ‘Willamette’ cultivar variety was selected for the analysis as it contained the highest concentrations of isoquercetin and quercetin 3-O-glucuronide.

Initially, the comprehensive LCxLC system was build with two columns: Nucleodur Sphinx RP column (50 mm × 1 mm, 5 μm) in the first dimension and Chromolith Flash RP-18e (25 mm × 4.6 mm) column in the second dimension. Nucleodur Sphinx RP column contains silica gel chemically modified with phenyl groups and octadecyl groups in a 1:1 ratio. The separation mechanism is based on both aromatic ring π-π interactions and hydrophobic interactions (C-18). However, the participation of phenyl groups in separation mechanism decreases with the increase of acetonitrile in the mobile phase. The separation in the first dimension was performed using gradient elution program II, of an increasing concentration of a mixture of TFA:acetonitrile:water (0.1:50:50, v/v/v), from 10% to 56%, in a TFA:water mixture (0.1:100, v/v). The fractions from the first column were automatically transferred to the second column, in modulation time of 2 min, and resolved using gradient elution program III, of an increasing concentration of a mixture of TFA:acetonitrile:water (0.1:50:50, v/v/v), from 0 to 55%, in a TFA:water solution (0.1:100, v/v), at mobile phase flow of 1.5 ml/min. As a further optimization of the LCxLC separation process, a longer column – Chromolith Performance (25 mm × 4.6 mm) was used in the second dimension instead of Chromolith Flash RP-18e. The same gradient program was maintained but the flow rate of the mobile phase was increased four times (Figure 3). A total of approximately 50 compounds were separated and visible as resolved spots on the obtained LCxLC chromatogram, including isoquercetin and quercetin 3-O-glucuronide (Figure 3). Other separated and identified compounds comprised caffeic acid, gallic acid, protocatechuic acid ellagic acid, catechin, epicatechin, procyanidin B_2_, sanguiin H-6, hyperoside and kaempferol 3-O-galactoside (Figure 3). The peaks corresponding to other compounds, including ellagitannins identified by HPLC-DAD-ESI-MS method were not shown on the LCxLC chromatogram due to their low concentration in the shoot extract.

### Antioxidant activity

The EC_50_ of the shoot extract from the ‘Willamette’ variety in the DPPH scavenging assay was established at 19.4 μg/ml and the reducing power in the phosphomolybdenum assay was AAE 427.94 mg/g. These values point out the excellent antioxidative properties of the shoot extract comparable to other plant sources of strong antioxidants, like *Potentilla* sp. (EC_50_ - 16.9 to 23.9 μg/ml) [30], rose (EC_50_ 12.24 μg/ml, AAE 354.87 mg/g) [31] representing *Rosaceae*, or peppermint, thyme, rosemary, and sage from *Lamiaceae* (EC_50_ 15–21 μg/ml, AAE 156–41 mg/g) [32, 33]. Raspberry shoot extract was also a much stronger DDPH scavenger than blackberry leaves extracts from three other *Rubus spp*. which had EC_50_ values higher than 180 μg/ml [34]. Similarly, some other plants tested with the same methods were several times less potent, e.g. parsley, dill, nettle, senna, and laurel leaves [35] or *Iris domestica* rhizomes [36]. Raspberry shoots also proved to have two times higher antoxidative capacities than the fruits of the black raspberry and about 10 times higher than red raspberries. The reducing power of the shoots was also about two to three times higher than in the raspberry fruits (unpublished results).

The high sanguiin H-6 content in the shoots is presumed to be the reason for the high antioxidative capacity of the *R. idaeus* ‘Willamette’ extract. This is in agreement with the findings about the antioxidative activity of raspberry fruits which confirm that ellagitannin-rich fractions containing sanguiin H-6 obtained from the fruits of *R. idaeus*, display stronger antioxidative capacities than the other, anthocyanin-containing fractions [7, 37, 38].

Taking into account the possible significance of antioxidants in inhibiting inflammation [39, 40] an anti-inflammatory assay was performed for the shoots using an in vivo rat model of carrageenan-induced paw edema [41, 42]. However no statistically significant anti-inflammatory properties were observed for the shoot extract (data not shown).

### Antimicrobial activity

The results of antimicrobial activity of *R. idaeus* ‘Willamette’ shoot extract, with ampicillin as a reference, are presented in Table 5. Among the tested strains varied sensitivities to the extracts were observed. Inhibitory activity was observed towards *Bacillus subtilis*, *Clostridium sporogenes*, *Staphylococcus epidermidis*, *Neisseria meningitidis*, *Moraxella catarrhalis* and *Helicobacter pylori* at concentrations ranging from 0.2 to 30 mg/ml. Bactericidal activity was observed for eight bacterial strains at concentrations ranging from as low as 0.06 mg/ml for *Corynebacterium diphtheriae* to the maximal tested concentration of 120 mg/ml for *E. faecalis* (Table 5).

**Table 5:** **Antimicrobial activity of** ***R. idaeus*** **‘Willamette’ shoot extract and ampicillin (mg/ml)**

Bacterial strain	***R. idaeus***‘Willamette’		Ampicilin	
	MIC	MBC	MIC	MBC
*Streptococcus* group A	3.75	7.5	0.3 × 10^−3^	0.3 × 10^−3^
*Streptococcus* group B	60	60	0.6 × 10^−3^	-
*Streptococcus* group G	15	15	0.2 × 10^−3^	-
*Streptococcus pneumoniae*	7.5	7.5	0.2 × 10^−3^	10 × 10^−3^
*Enterococcus faecalis*	120	120	2.5 × 10^−3^	2.5 × 10^−3^
*Corynebacterium diphtheriae*	0.06	0.06	0.2 × 10^−3^	0.3 × 10^−3^
*Bacillus subtilis*	30	-	0.05 × 10^−3^	0.3 × 10^−3^
*Clostridium sporogenes*	0.2	-	1 × 10^−3^	-
*Staphylococcus aureus*	0.5	1.0	0.1 × 10^−3^	0.2 × 10^−3^
*Staphylococcus epidermidis*	1.9	-	0.6 × 10^−3^	0.3 × 10^−3^
*Neisseria meningitidis*	30	-	0.2 × 10^−3^	10 × 10^−3^
*Moraxella catarrhalis*	0.5	-	2.5 × 10^−3^	2.5 × 10^−3^
*Haemophilus influenzae*	-	-	0.2 × 10^−3^	0.2 × 10^−3^
*Helicobacter pylori*	7.4	-	3.3 × 10^−3^	3.3 × 10^−3^
*Klebsiella pneumoniae*	60	60	62.5 × 10^−3^	-

MIC - minimal inhibitory concentration; MBC - minimal bactericidal concentration.

Interestingly, *C. diphtheriae* proved to be the most sensitive bacterium displaying MBC values at the lowest tested concentration of 0.06 mg/ml. *Staphylococcus aureus* was another very sensitive bacterium with both MIC and MBC below 1 mg/ml (0.47 and 0.94 mg/ml respectively). The extract also displayed strong inhibitory activity towards *C. sporogenes* (0.23 mg/ml) and *M. catarrhalis* (0.47 mg/ml). The only bacterium resistant to the shoot extract was *H. influenzae* (Table 5). These results are in accordance with our findings about the antimicrobial properties of raspberry fruit extracts where *C. diphtheriae*, *S. aureus*, *M. catarrhalis* and *C. sporogenes* were the most sensitive bacteria, although the shoot extract stands out as a more potent inhibitory and bactericidal agent compared to the fruit extracts (accepted paper).

The antimicrobial properties of raspberry shoots have not been studied but the antimicrobial properties of raspberry fruits is the subject of a few papers and their antimicrobial potential is linked mainly to the presence of ellagitannins [38, 43–46]. As the raspberry shoot extract is a prospective source of sanguiin H-6 and ellagic acid, these two components are believed to be the ones responsible for its antimicrobial properties.

### Cytotoxic activity

The *in vitro* cytotoxicity of *R. idaeus* ‘Willamette’ shoot extract and sanguiin H-6 was tested using human promyelocytic leukemia cell line (HL-60), human cervical cancer cell line (HeLa) and human dermal fibroblasts. The IC_50_ values for the respective cell lines are presented in Table 6. The strongest cytotoxic activity of the extract was observed for the HL-60 cells (110 μg/ml). Sanguiin H-6 also displayed distinctive cytotoxic activity in HeLa (35 μg/ml) and HL-60 (25 μg/ml) cell lines. No cytotoxic activity was observed in the human fibroblasts. The cytotoxic activity of the shoot extract can be related to its high sanguiin H-6 content as well. The findings of Ross et al. [37] confirm that the antiproliferative effect of raspberry fruit extracts towards the HeLa cell lines is predominantly associated with the presence of ellagitannins.

**Table 6:** **Cytotoxic activity of** ***R. idaeus*** **‘Willamette’ shoot extract and sanguiin H-6 (IC**_**50**_ **, μg/ml) (n = 3)**

	HeLa	HL-60	Fibroblasts
*R. idaeus* ‘Willamette’	300 ± 23	110 ± 5.5	-
Sanguiin H-6	35 ± 1.4	25 ± 1.2	-

## Conclusions

The obtained results are the first to present the chemical composition of the shoots *of R. idaeus*. They show the occurrence of various phenolic compounds, including simple phenols like ellagic acid, and polyphenols such as ellagitannins (sanguiin H-6) and flavonoids. The shoots of *R. idaeus* stand out as a valuable and selective source of sanguiin H-6 and ellagic acid and reveal a number of biological properties including antimicrobial, antioxidative and cytotoxic activity.
